# Automated Cell-Free Multiprotein Synthesis Facilitates the Identification of a Secretory, Oligopeptide Elicitor-Like, Immunoreactive Protein of the Oomycete Pythium insidiosum

**DOI:** 10.1128/mSystems.00196-20

**Published:** 2020-05-12

**Authors:** Pattarana Sae-Chew, Thidarat Rujirawat, Yothin Kumsang, Penpan Payattikul, Tassanee Lohnoo, Wanta Yingyong, Chalisa Jaturapaktrarak, Tiwa Rotchanapreeda, Onrapak Reamtong, Tanawut Srisuk, Weerayuth Kittichotirat, Theerapong Krajaejun

**Affiliations:** a Research Center, Faculty of Medicine, Ramathibodi Hospital, Mahidol University, Bangkok, Thailand; b Department of Pathology, Faculty of Medicine, Ramathibodi Hospital, Mahidol University, Bangkok, Thailand; c Department of Molecular Tropical Medicine and Genetics, Faculty of Tropical Medicine, Mahidol University, Bangkok, Thailand; d Systems Biology and Bioinformatics Research Group, Pilot Plant Development and Training Institute, King Mongkut’s University of Technology Thonburi, Bangkhuntien, Bangkok, Thailand; UiT—The Arctic University of Norway

**Keywords:** *Pythium insidiosum*, cell-free protein synthesis, evolution, immunoreactive protein, oomycete, pythiosis, oligopeptide elicitor

## Abstract

Protein production relies on time-consuming genetic engineering and *in vivo* expression, which is a bottleneck for functional studies in the postgenomic era. Cell-free protein synthesis (CFPS) overcomes the limitation of *in vivo* protein biosynthesis by processing *in vitro* transcription and translation of multiple genes to proteins within hours. We employed an automated CFPS to simultaneously synthesize proteins from 24 genes of the oomycete Pythium insidiosum (which causes the life-threatening disease pythiosis) and screen for a diagnostic and therapeutic target. CFPS successfully synthesized 18 proteins (∼75% success rate). One protein, namely, I06, was explicitly recognized by all pythiosis sera, but not control sera, tested. *Py. insidiosum* secreted a significant amount of I06. The protein architecture of I06 is compatible with the oligopeptide elicitor (OPEL) of the phylogenetically related plant-pathogenic oomycete Phytophthora parasitica. The OPEL-like I06 protein of Py. insidiosum can stimulate host antibody responses, similar to the P. parasitica OPEL that triggers plant defense mechanisms. OPEL-like I06 homologs are present only in the oomycetes. *Py. insidiosum* contains two OPEL-like I06 homologs, but only one of the two homologs was expressed during hyphal growth. Twenty-nine homologs derived from 15 oomycetes can be phylogenetically divided into two groups. The OPEL-like genes might occur in the common ancestor, before independently undergoing gene gain and loss during the oomycete speciation. In conclusion, CFPS offers a fast *in vitro* protein synthesis. CFPS simultaneously generated multiple proteins of *Py. insidiosum* and facilitated the identification of the secretory OPEL-like I06 protein, a potential target for the development of a control measure against the pathogen.

**IMPORTANCE** Technical limitations of conventional biotechnological methods (i.e., genetic engineering and protein synthesis) prevent extensive functional studies of the massive amounts of genetic information available today. We employed a cell-free protein synthesis system to rapidly and simultaneously generate multiple proteins from genetic codes of the oomycete Pythium insidiosum, which causes the life-threatening disease called pythiosis, in humans and animals worldwide. We aimed to screen for potential diagnostic and therapeutic protein targets of this pathogen. Eighteen proteins were synthesized. Of the 18 proteins, one was a secreted immunoreactive protein, called I06, that triggered host immunity and was recognized explicitly by all tested sera from pythiosis patients. It is one of the OPEL proteins; these proteins are present only in the unique group of microorganisms called oomycetes. Here, we demonstrated that cell-free protein synthesis was useful for the production of multiple proteins to facilitate functional studies and identify a potential target for diagnosis and treatment of pythiosis.

## Abstract (precis)

Technical limitations of conventional biotechnological methods (i.e., genetic engineering and protein synthesis) prevent extensive functional studies of the massive amounts of genetic information available today. We employed a cell-free protein synthesis system to rapidly and simultaneously generate multiple proteins from genetic codes of the oomycete Pythium insidiosum, which causes the life-threatening disease called pythiosis, in humans and animals worldwide. We aimed to screen for potential diagnostic and therapeutic protein targets of this pathogen. Eighteen proteins were synthesized. Of the 18 proteins, one was a secreted immunoreactive protein, called I06, that triggered host immunity and was recognized explicitly by all tested sera from pythiosis patients. It is one of the OPEL proteins; these proteins are present only in the unique group of microorganisms called oomycetes. Here, we demonstrated that cell-free protein synthesis was useful for the production of multiple proteins to facilitate functional studies and identify a potential target for diagnosis and treatment of pythiosis.

## INTRODUCTION

Pythium insidiosum belongs to the unique group of fungus-like eukaryotic microorganisms called oomycetes. It causes pythiosis, a life-threatening disease in humans and other animals, including horses, dogs, cats, and cattle (1). The treatment of pythiosis is challenging. Conventional antifungal drugs and vaccine immunotherapy provide limited efficacy against pythiosis (2–4). To control the infection, many patients undergo removal of the affected organ, such as enucleation and limb amputation (5–7). A fatal outcome is inevitable in pythiosis patients with advanced disease. We urgently need an effective noninvasive treatment for the infection caused by this understudied pathogen.

A better understanding of the pathophysiology of Py. insidiosum could lead to the identification of a suitable target for the future development of a potent drug or vaccine. Next-generation sequencing technology has emerged and become a standard platform for generating genomic data for many organisms, including nonmodel microorganisms like *Py. insidiosum*. Our group has reported the first draft genome sequence of *Py. insidiosum*, which contains a total of 14,962 predicted open reading frames (ORFs) (8). Mass spectrometric analysis of crude protein extract of *Py. insidiosum* can validate the expression in 4,445 out of these ORFs (9). Such genomic and proteomic data can now serve as an invaluable resource for exploring the biology and pathogenicity of *Py. insidiosum*. The next challenging step is to elucidate the roles of these genes.

A functional study of an uncharacterized gene could begin with protein expression. A commonly used method to produce a recombinant protein relies on genetic engineering and *in vivo* biosynthesis using a host cell of choice, such as the bacterium Escherichia coli. Such an approach requires several time-consuming, laborious, and complicated steps, including (i) molecular cloning of a protein-coding sequence into an appropriate plasmid DNA vector, (ii) transforming the vector into the desired host, and (iii) optimizing the protein expression condition (i.e., incubation time, temperature, and protein isolation and purification) (10, 11). Furthermore, some proteins possess cytotoxicity to the host cell and require additional procedures, such as coupling and splicing of a fusion protein for the downstream purification step (12, 13). Thus, the generation of only one recombinant protein could take months, which is a bottleneck for functional analyses in the postgenomic era, where a vast amount of genetic information is readily available.

A cell-free protein synthesis system (CFPS) could overcome the limitation of conventional cell-based protein synthesis because it can produce multiple proteins that can then be purified within hours (14, 15). CFPS incorporates *in vitro* transcription and translation of a protein-coding sequence in the form of either PCR product or plasmid DNA and employs an E. coli lysate containing the components necessary for protein synthesis (i.e., T7 RNA polymerase, ribosome, tRNA, and energy source) and buffers supplemented with amino acids and nucleoside triphosphates (NTPs) (16–19). In the current study, we employed a commercially available automated CFPS to generate multiple proteins of *Py. insidiosum*. From a total of 24 PCR-amplified protein-coding sequences randomly selected from the draft genome of *Py. insidiosum*, 18 were successfully expressed by CFPS. One gene product (assigned as I06) was characterized as a secretory, oomycete-specific, oligopeptide elicitor (OPEL)-like protein. It was an immunoreactive protein that could be a potential diagnostic and therapeutic target of *Py. insidiosum*.

## RESULTS

### Gene selection and amplification for cell-free protein synthesis.

**FIG 1:**
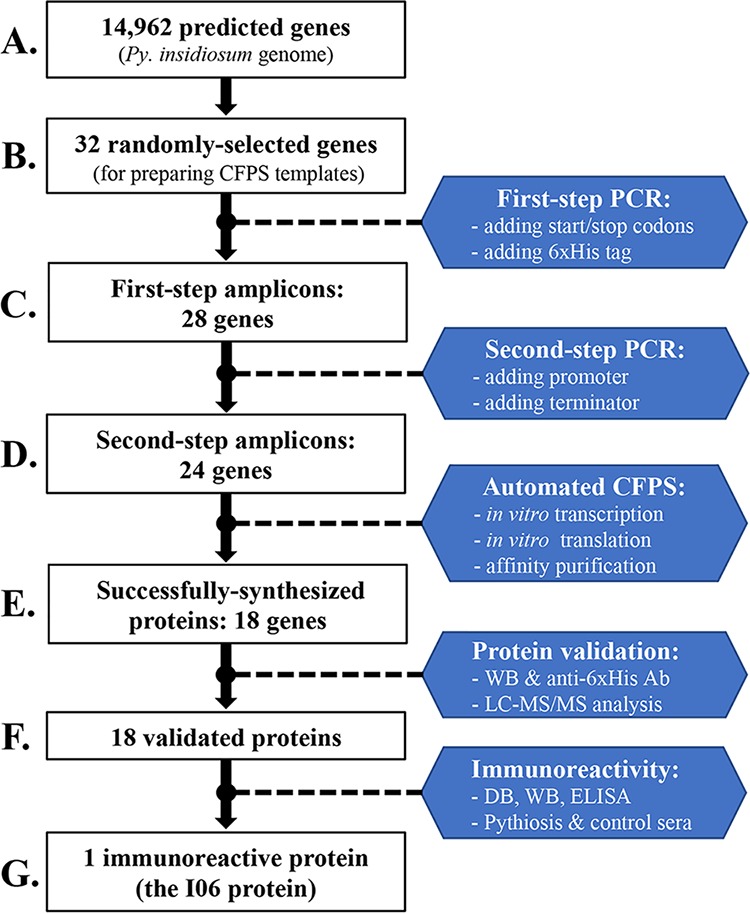
Cell-free multiprotein synthesis workflow used in this study. (A) A total of 14,962 genes are predicted in the genome of Pythium insidiosum. (B) Thirty-two genes are randomly selected for the cell-free protein synthesis (CFPS) template preparation. (C) First-step PCR successfully amplifies 28 target genes from the *Py. insidiosum* genomic DNA using the gene-specific primers tagged with adaptor sequences (i.e., start and stop codons and 6×His tag). (D) Second-step PCR can add the ribosome binding site, promoter, and terminator sequences to 24 genes (first-step amplicons) using the CFPS general primers. (E) Automated CFPS system generates and purifies synthesized proteins from 18 target genes by employing *in vitro* transcription and translation using E. coli-derived reagents (∼3 h) and affinity-based protein purification (∼3 h). (F) All 18 synthesized proteins can be validated by Western blotting (WB) (using the anti-6×His tag antibody [Ab]) and liquid chromatography-tandem mass spectrometry (LC-MS/MS) analysis. (G) Eighteen synthesized proteins are screened for immunoreactivity against a set of 21 pythiosis and 25 control sera, using dot blotting (DB), WB, and enzyme-linked immunosorbent assay (ELISA). Only one synthesized protein, namely, I06, is recognized by all pythiosis sera, but not any control sera, tested.

The overall process of cell-free protein synthesis was summarized in Fig. 1. From a total of 14,962 genes predicted in the *Py. insidiosum* genome (8), 32 were randomly selected for the cell-free protein synthesis (Fig. 1A and B; see also Table S1 in the supplemental material). Most deduced proteins (*n* = 22) can be functionally annotated, while the rest (*n* = 10) were assigned as hypothetical proteins. Liquid chromatography-tandem mass spectrometry (LC-MS/MS) data generated from soluble antigen from broken hyphae (SABH) (representing the cytosolic proteins) of *Py. insidiosum* (9) can validate the expression of all, except three (identifiers [IDs] 7, 8, and I03), deduced proteins (Table S1).

10.1128/mSystems.00196-20.5 TABLE S1 List of 32 coding sequences from *Py. insidiosum* for cell-free protein synthesis. Functional description, accession number, primer sequences, PCR result, and protein product of each gene are shown in the table. Download Table S1, PDF file, 0.1 MB. Copyright © 2020 Sae-Chew et al. 2020 Sae-Chew et al. This content is distributed under the terms of the Creative Commons Attribution 4.0 International license.

Gene-specific primers were designed to amplify a full-length coding sequence of 12 genes (containing no intron; amplicon sizes, 519 to 2,850 bp) and a partial coding sequence of 20 genes (including at least one intron; amplicon sizes, 489 to 2,046 bp) (Table S1). The generation of a coding sequence template for cell-free protein synthesis was depicted in Fig. S1 in the supplemental material. Of 32 selected genes, 28 genes were successfully amplified (Fig. 1C; Fig. S2A) by the first-step PCR using the *Py. insidiosum* genomic DNA (gDNA) (as the template) and the gene-specific primers attached with either the forward (containing a start codon and 6×His tag) or reverse (containing a stop codon) adaptor (Fig. S1A). Although the manufacturer recommends an annealing temperature of 58°C, optimization of the temperature was required for some genes (IDs, 2, 4, 7, I13, I14, I18, and I21) to achieve a better-quality PCR product (Table S1). As a result, a prominent PCR product was obtained from 24 genes, whereas a few faint amplicons were observed for 4 genes (IDs, 2, 7, I13, and I21; Fig. S2A). All first-step amplicons from 28 genes served as the template of the second-step PCR (Fig. S1B), which added 200-bp upstream and downstream cassette sequences that contain the genetic components required for protein expression (Fig. S1C). An expected prominent PCR product was obtained from 24 genes (Fig. 1D; Fig. S2B). DNA sequencing proved that these 24 gel-purified amplicons contained the correct protein-coding sequences. Several faint bands were observed in the other four genes (IDs, 2, 7, I13, and I21) (Fig. S2B), which were excluded from the cell-free protein synthesis.

10.1128/mSystems.00196-20.1 FIG S1 Preparation of a protein-coding sequence template for cell-free protein synthesis. (A) First-step PCR amplifies a target gene from the gDNA template, using the gene-specific primers (1F and 1R) attached with the adaptor sequence containing start codon, 6×-His tag, or stop codon. (B) The resulting amplicon serves as the second-step PCR template for adding the upstream and downstream cassette sequences that contain T7 promoter, ribosomal binding site (RBS), or T7 terminator. (C) The final PCR product, amplified by using primers 2F and 2R, is used as a formatted protein-coding sequence template for cell-free protein synthesis. Download FIG S1, TIF file, 2.3 MB. Copyright © 2020 Sae-Chew et al. 2020 Sae-Chew et al. This content is distributed under the terms of the Creative Commons Attribution 4.0 International license.

10.1128/mSystems.00196-20.2 FIG S2 Preparation of formatted protein-coding sequence templates from the *Py. insidiosum* genes. (A) First-round PCR amplification provides expected amplicons from 28 out of 32 selected genes. (B) Second-round PCR amplification successfully adds the 200-bp upstream and downstream cassette sequences to 24 first-round amplicons to serve as the formatted protein-coding sequence templates. The molecular sizes (100-bp step ladder) are shown to the left of the gel. Download FIG S2, TIF file, 0.5 MB. Copyright © 2020 Sae-Chew et al. 2020 Sae-Chew et al. This content is distributed under the terms of the Creative Commons Attribution 4.0 International license.

### Automated cell-free synthesis and validation of *Py. insidiosum* proteins.

Up to 16 proteins (per run) were simultaneously synthesized from all 24 expected-size second-step amplicons, using a commercially available cell-free protein synthesis kit (Bioneer). An automated protein synthesis machine (Bioneer) was employed to finish two processes: (i) transcription and translation of a coding sequence to a protein (∼3 h); and (ii) affinity-based purification of an obtained product to a ready-to-use protein (∼3 h). Sodium dodecyl sulfate-polyacrylamide gel electrophoresis (SDS-PAGE) and Western blot analyses were used to check for the presence of a protein. Successful protein synthesis was observed in 18 genes, providing protein concentrations of 92 to 387 μg/ml (Fig. 1E; Fig. S3; Table S1). Five background proteins (with sizes of 75, 25, 20, 15, and 10 kDa) from the E. coli extract were present in all samples, including the green fluorescent protein (GFP) (positive control). Fourteen genes expressed a single protein (with expected size), while the others (IDs, 8, 11, I02, and I19) provided multiple gene products. Based on Western blot analysis (Fig. 1E and F), the mouse anti-6×His tag antibody correctly recognized 16 synthesized proteins at the expected size (Fig. S3). In contrast, the antibody did not show any immunoreactivity against two other proteins (IDs, 1 and 8).

10.1128/mSystems.00196-20.3 FIG S3 Identification of synthesized proteins of *Py. insidiosum* by Western blot analysis. Eighteen synthesized proteins tagged with polyhistidine (6×-His) are separated by SDS-PAGE and blotted on a nitrocellulose membrane. The separated proteins are probed with the mouse anti-6×-His monoclonal antibody. Protein ID and size (kDa) are indicated in each lane. Molecular weight markers (ranging from 20 to 100 kDa) are shown to the left of the membrane. Download FIG S3, TIF file, 1.0 MB. Copyright © 2020 Sae-Chew et al. 2020 Sae-Chew et al. This content is distributed under the terms of the Creative Commons Attribution 4.0 International license.

**FIG 2:**
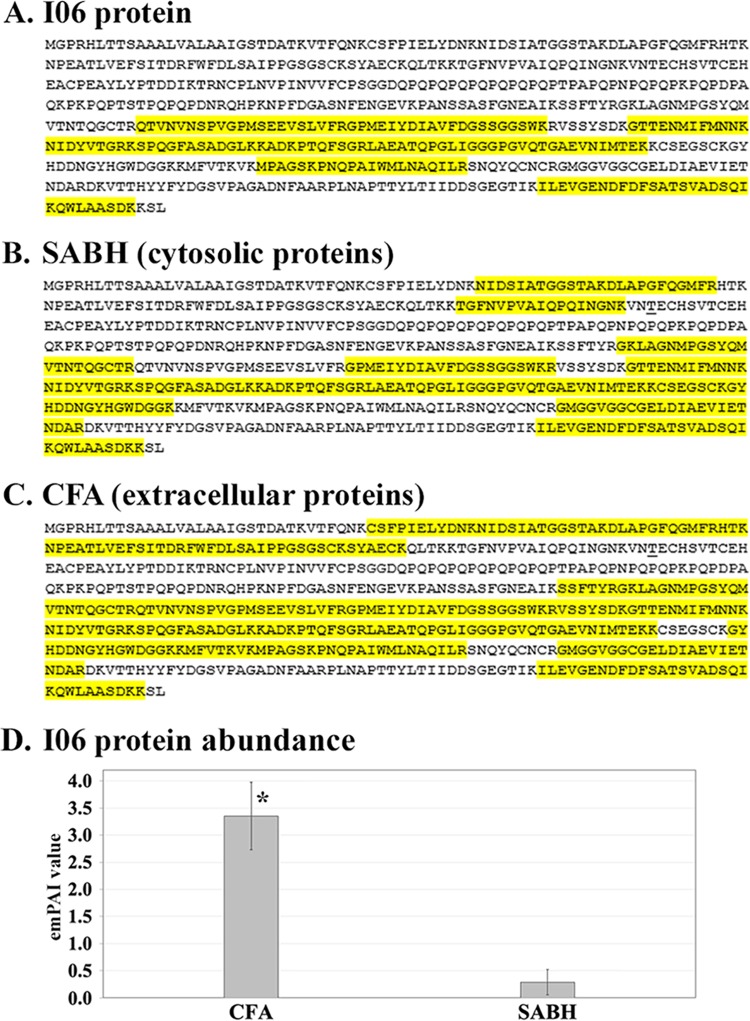
Validation, cellular localization, and abundance of the I06 protein of *Py. insidiosum*. (A to C) LC-MS/MS-generated data from the synthesized I06 protein (A), soluble antigens from broken hyphae (SABH) (representing cytosolic proteins) (B), and culture filtrate antigen (CFA) (representing extracellular proteins) (C) can map 12, 15, and 32 peptides of the I06 protein, respectively (labeled yellow). (D) The LC-MS/MS data of the I06 protein were quantitatively transformed into the exponentially modified protein abundance index (emPAI). Based on the independent *t* test with 95% confidence, the emPAI value of the I06 protein in CFA was significantly higher than that of the I06 protein in SABH (the asterisk indicates *P* value of <0.05). Error bars represent the standard error of the mean (SEM) of each group.

For further protein validation (Fig. 1E and F), all 18 synthesized proteins were excised from the SDS-PAGE gel, tryptic digested, and proceeded with the LC-MS/MS analysis against the in-house Mascot library of 14,962 *Py. insidiosum* proteins (9). As a result, the sequences of all 18 synthesized proteins can map their corresponding LC-MS/MS-generated peptides (average number of mapped peptides per protein, ~14; range, 3 to 37) (Table S1). The synthesized I06 protein (which was selected for further characterization) matched 12 different LC-MS/MS-generated peptides (Fig. 2A).

### Immunoreactivity of the synthesized proteins of *Py. insidiosum*.

**FIG 3:**
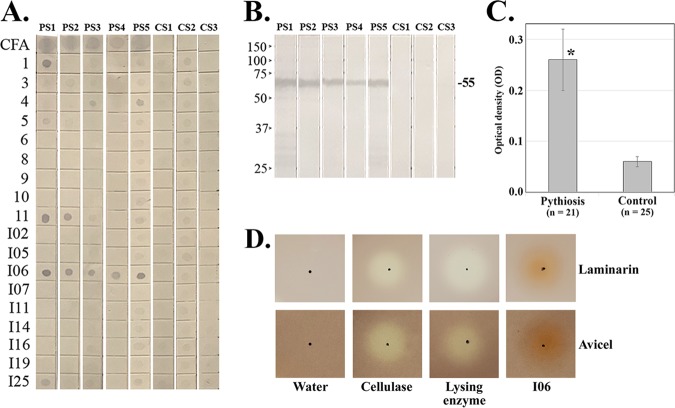
Immunoreactivity of synthesized proteins of *Py. insidiosum*. (A) Eighteen synthesized proteins are spotted on a nitrocellulose membrane and probed with serum samples from pythiosis patients (*n* = 5; PS1 to PS5) and healthy blood donors (served as control; *n* = 3; CS1 to CS3). (B) The 55-kDa I06 protein is separated by SDS-PAGE and blotted on a nitrocellulose membrane, before probing with the pythiosis (PS1 to PS5) and control (CS1 to CS3) sera (molecular weight markers [in kilodaltons] are shown to the left of the membrane). (C) Protein A/G-based ELISA evaluates the immunoreactivity of the I06 protein against a panel of 21 pythiosis and 25 control serum samples from humans and various animals. The mean optical density (OD) of the pythiosis sera is significantly higher than that of the control sera (the asterisk indicates a *P* value of <0.01). Error bars represent the standard error of the mean (SEM) of each group. (D) Agar plate enzymatic assay for assessment of glycoside hydrolase activity of distilled water (negative control), Trichoderma reesei cellulase (positive control), *Trichoderma harzianum* lysing enzyme (positive control), and synthesized I06 protein against two substrates, Avicel (microcrystalline cellulose) and laminarin. The dark spot indicates the position where the water, cellulase, lysing enzyme, or I06 protein was applied.

Dot blot analysis was used to screen the immunoreactivity of all 18 synthesized proteins and culture filtrate antigen (CFA) (crude protein extract; served as positive control) of *Py. insidiosum* against pythiosis (sera from five patients; samples PS1 to PS5) and control (sera from three healthy blood donors; samples CS1 to CS3) serum samples. CFA showed strong immunoreactivity against all pythiosis sera, but not the control sera (Fig. 3A). Six synthesized proteins (IDs, 1, 4, 5, 11, I06, and I25) exhibited prominent immunoreactivity against at least one pythiosis serum but showed a modest signal or no signal to all control sera (Fig. 3A). Only one synthesized protein (ID, I06) was strongly recognized by all pythiosis sera tested (Fig. 1G). Western blot analysis confirmed that the 55-kDa I06 protein strongly immunoreacted with the pythiosis sera, but not the control sera (Fig. 3B). The immunoreactivity of the I06 protein was further investigated by using an enzyme-linked immunosorbent assay (ELISA) (20, 38) and an extended number of pythiosis (*n* = 21) and control (*n* = 25) sera from various hosts (i.e., 32 humans, 8 horses, 4 dogs, 1 cat, and 1 cow) (Fig. 3C). The average optical density (OD) of the pythiosis sera (mean, 0.26; standard error of the mean [SEM], 0.06) was significantly higher than that of the control sera (mean, 0.06; SEM, 0.01) (*P* value of <0.01).

### I06 of *Py. insidiosum* is a secreted OPEL-like protein.

**FIG 4:**
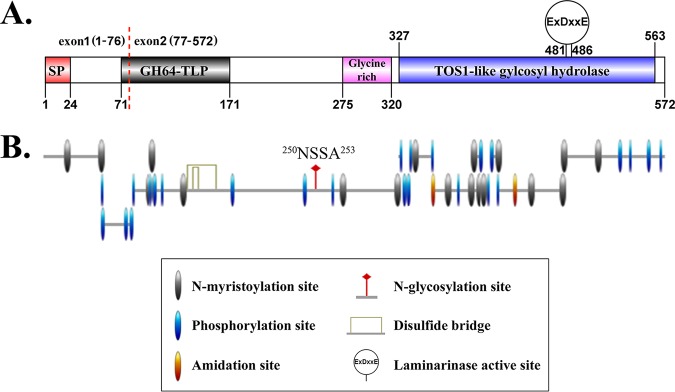
Architecture and posttranslational modification of the I06 protein of *Py. insidiosum*. (A) The full-length I06 protein (572 amino acids long) contains a signal peptide (SP), a glycoside hydrolase family 64 and thaumatin-like protein domain (GH64-TLP), a glycine-rich protein domain, and a TOS1-like glycosyl hydrolase domain with one laminarinase active site “ExDxxE” (x represents any amino acid). The red dashed line divides the I06 protein into a small portion (amino acid residues 1 to 76) derived from exon 1 and a large portion (amino acid residues 77 to 572) derived from exon 2. (B) Putative posttranslational modifications of the I06 protein include N-myristoylation, protein kinase C phosphorylation, casein kinase II phosphorylation, cyclic AMP (cAMP)- and cGMP-dependent protein kinase phosphorylation, amidation site, N-glycosylation site, and disulfide bridge.

Genome and transcriptome data of Py. insidiosum (8, 21) and gene model prediction (9) indicated that the I06 protein-encoding gene was 1,790 bases long and consisted of two exons (base positions, 1 to 226 for exon 1 and 298 to 1790 for exon 2). The full-length deduced protein was 572 amino acids long with an estimated molecular mass of 61.5 kDa and pI of 6.12. The protein contained a signal peptide (amino acid positions, 1 to 24), a glycoside hydrolase family 64 and thaumatin-like protein domain (GH64-TLP; positions, 71 to 171), a glycine-rich protein domain (positions, 275 to 320), and a TOS1-like glycosyl hydrolase domain (positions, 327 to 563) with one laminarinase active site “ExDxxE” (x represents any amino acid) (22); positions, 481 to 486) (Fig. 4A). The ScanProsite program predicted two disulfide bridges and several posttranslational modification types, such as N-myristoylation, phosphorylation, amidation, and N-glycosylation (Fig. 4B).

The nearly complete I06 protein (503 amino acids long, including a polyhistidine [6×His] tag; 55 kDa in size) was synthesized from exon 2 (excluding the first 76 N-terminal amino acids of exon 1) (Fig. S3 and Fig. 3B). The mass spectrometric analysis was used to investigate the cellular location of the I06 protein. LC-MS/MS-derived peptides from SABH (representing cytosolic proteins) (9) and CFA (representing extracellular proteins; unpublished data) of *Py. insidiosum* can map 15 and 32 different peptides of the I06 protein, respectively (Fig. 2B and C). Glycoside hydrolase activity of the synthesized I06 protein, which contains a putative TOS1-like glycosyl hydrolase domain with one laminarinase active site (Fig. 4A), was assessed using the agar plate enzymatic assay (Fig. 3D), as described previously (23, 24). Two positive controls (i.e., Trichoderma harzianum lysing enzyme and Trichoderma reesei cellulase) showed a hydrolytic zone on agar containing either Avicel (microcrystalline cellulose; 0.9- and 1.0-cm clear zone, respectively) or laminarin (1.1- and 1.0-cm clear zone, respectively). In contrast, the synthesized I06 protein did not show any clear zone on both agar types.

### I06 homologs are present only in the oomycetes and divided into two phylogenetic groups.

**FIG 5:**
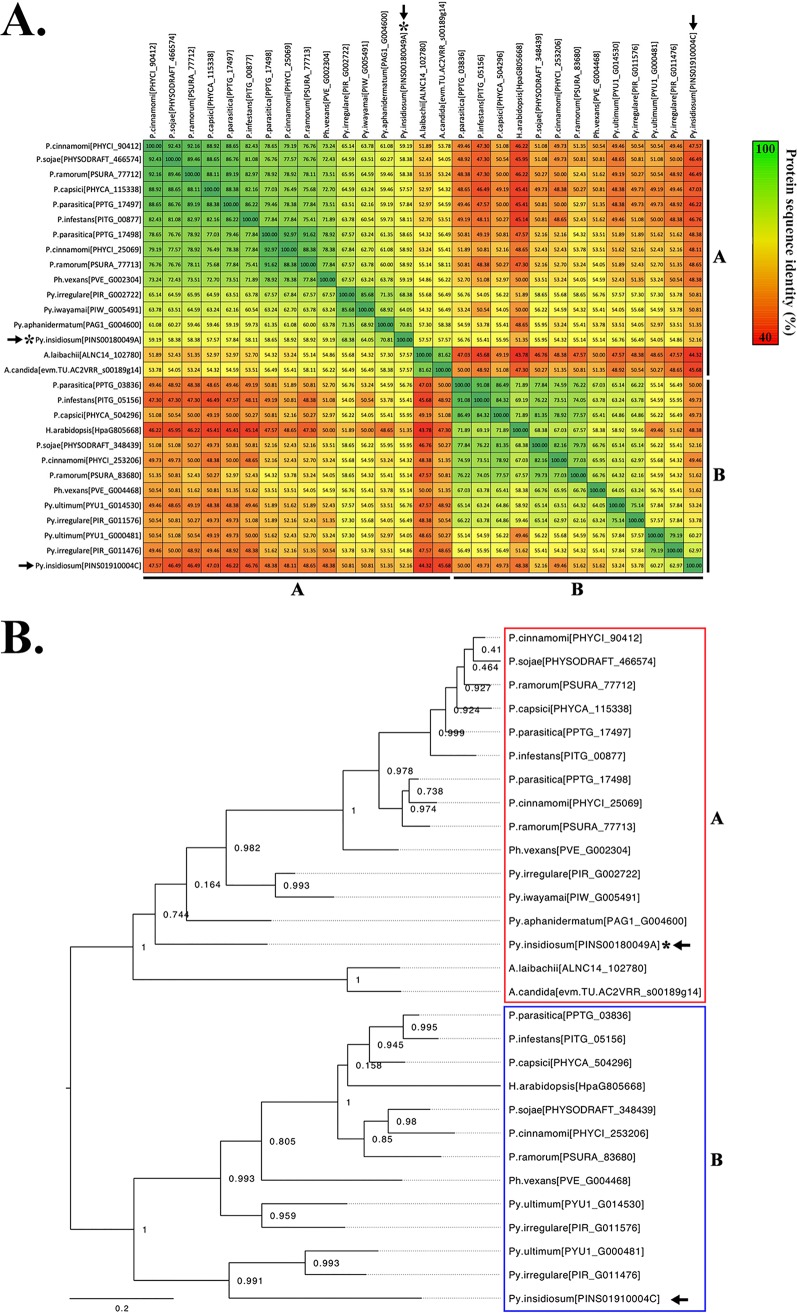
Comparison and phylogenetic analysis of 29 OPEL-like I06 homologs from 15 oomycetes. (A) Pairwise comparison of the OPEL-like I06 homologs. The protein ID of each homolog is present in the brackets after the organism’s name. Protein sequence similarities are presented as a percentage, as shown by gradient colors ranging from 44% (dark red) to 100% (dark green). The arrows indicate both I06 homologs of *Py. insidiosum* allocated in phylogenetic groups A and B (see Fig. 5B). The asterisk marks the prototype I06 protein from *Py. insidiosum*. (B) Maximum likelihood phylogenetic tree generated from the OPEL-like I06 homologs. All I06 homologs are classified into two groups, groups A (16 homologs) and B (13 homologs). The bootstrap-based reliability of the tree to support each branch is shown.

A BLAST search using the I06 protein sequence against the National Center for Biotechnology Information (NCBI) and FungiDB databases identified no homolog in non-oomycete organisms, including humans and fungi. By searching through the Oomycete Gene Table containing the genome contents of 20 oomycetes (including *Py. insidiosum*) and 2 diatoms (9, 25), 29 homologs of the I06 protein were identified in 15 oomycetes (up to 3 homologs per species) (Fig. S4). All identified homologs were grouped in the cluster “p-cluster 053361” defined by the Oomycete Gene Table. Five oomycetes (namely, Pythium iwayamai, Pythium aphanidermatum, Albugo laibachaii, Albugo candida, and Hyaloperonospora arabidopsis) had only one copy of the I06 homolog. Four oomycetes (Pythium arrhenomanes, Aphanomyces astaci, Saprolegnia declina, and Saprolegnia parasitica) contained only a similar I06-coding sequence in their genomes and were excluded from the downstream analysis. No homolog was found in the oomycete Aphanomyces invadans and both diatoms (Phaeodactylum tricornutum and Thalassiosira pseudonana). Protein sequence identities of these I06 homologs (excluding self-again-self comparison) ranged from 44% to 93% (average, 59%), as showed by the pairwise comparison (Fig. 5A). Conserved domain analysis (26–29) indicated that the I06 homologs of *Py. insidiosum* and other oomycetes (except one of the Phytophthora vexans homologs) sequentially contained (from the N terminus to the C terminus) one each of signal peptide, GH64-TLP domain, glycine-rich domain, and TOS1-like glycosyl hydrolase domain (Fig. 4A), which is the typical characteristic of an oligopeptide elicitor or OPEL, previously described in Phytophthora parasitica (30).

10.1128/mSystems.00196-20.4 FIG S4 Copy number of genes encoding the OPEL-like I06 homolog and core proteins of oomycetes and diatoms. The Oomycete Gene Table shows a set of 29 genes encoding the OPEL-like I06 homologs found in 15 oomycetes (each species contains up to three copies, as indicated by the color), and a set of 14 single-copy core genes found in each oomycete. Gene cluster IDs and their associated functional descriptions are listed on the left. The arrow indicates the gene cluster ID that contains OPEL-like I06 homologs. The asterisk marks *Py. insidiosum*. A gray cell represents a sequence similar to that of the OPEL-like I06 homolog. A black cell indicates that neither OPEL-like I06 homolog nor a similar sequence is found. Download FIG S4, TIF file, 2.3 MB. Copyright © 2020 Sae-Chew et al. 2020 Sae-Chew et al. This content is distributed under the terms of the Creative Commons Attribution 4.0 International license.

**FIG 6:**
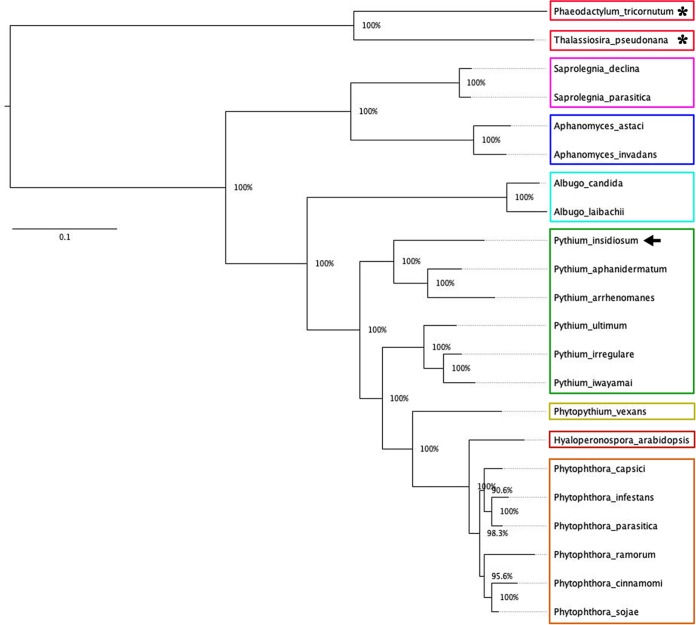
Core protein-based phylogenetic relationship of oomycetes. The maximum likelihood phylogenetic tree is generated using 14 core proteins identified across all 20 oomycetes and diatoms (assigned as the outgroup and indicated by asterisks). Functional descriptions of core proteins are listed in Fig. S4 in the supplemental material. The bootstrap-based reliability of the tree is shown to support each branch. The arrow indicates the phylogenetic position of *Py. insidiosum*.

The phylogenetic relationship of all organisms was analyzed using 14 single-copy core proteins (presented across 20 oomycetes and 2 diatoms; all proteins were concatenated to make one continuous sequence) and 29 homologs of the I06 protein (presented in 15 oomycetes) identified in the Oomycete Gene Table (Fig. S4). Based on the 14 single-copy core proteins, all oomycetes and diatoms were differentiated according to their lineages, as expected (Fig. 6). For example, while the diatoms served as an outgroup, *Py. insidiosum* was more closely related to the oomycetes of the genera *Pythium*, *Phytopythium*, *Phytophthora*, and *Hyaloperonospora* than those of the genera *Saprolegnia*, *Aphanomyces*, and *Albugo*. The phylogenetic analysis classified the I06 homologs into two groups, groups A (*n* = 16) and B (*n* = 13) (Fig. 5B). Nine oomycetes harboring multiple I06 homologs had their proteins allocated in both groups. However, six oomycetes (most of which, except Pythium ultimum, harbored a single-copy I06 homolog) selectively allocated their proteins in either group A (i.e., Py. iwayamai, Py. aphanidermatum, A. laibachaii, and A. candida) or group B (i.e., Py. ultimum and H. arabidopsis). Sequence identities of the I06 homologs within groups A and B (excluding self-again-self comparison) ranged from 51 to 93% (average, 68%) and 48 to 91% (average, 64%), respectively. Cross analysis of the I06 homologs in group A against that in group B showed sequence identities ranged from 44 to 59% (average, 51%).

By focusing on the two I06 homologs identified in *Py. insidiosum*, the Ion Torrent transcriptome analysis (unpublished data) showed that 1,110 transcript reads (per million reads that mapped to all *Py. insidiosum* genes) could match only the group A I06 homolog, while no generated transcript mapped the group B homolog. Likewise, the LC-MS/MS data generated from SABH (cytosolic protein extract) (9) and CFA (extracellular protein extract; unpublished data) mapped 15 and 32 peptides of the I06 protein in group A, respectively (Fig. 2B and C). No LC-MS/MS data mapped any peptides of the other I06 homolog in group B. The abundance of a protein of interest can be calculated based on the exponentially modified protein abundance index (emPAI) (9, 31, 32). The LC-MS/MS data of the I06 protein (from four biological replicates) were quantitatively transformed into emPAI. Based on the independent *t* test with 95% confidence, the emPAI value of the I06 protein in CFA (mean, 3.35; range, 1.59 to 4.61) was significantly (11.6-fold) higher than that of the I06 protein in SABH (mean, 0.29; range, 0.05 to 0.52) (Fig. 2D).

## DISCUSSION

CFPS was introduced in the 1950s and has employed the cellular fraction from a variety of organisms, such as rat liver (33), E. coli (34), wheat germ (35, 36), and rabbit reticulocyte (37). An automated CFPS (as used in this study) relies on the *in vitro* transcription and translation processes of the DNA template (PCR product or plasmid). The system contains the enzymes and other components prepared from E. coli and the exogenously supplied master mix containing NTPs, amino acids, energy sources, and salts. CFPS can be carried out in separate reaction tubes, which allow the simultaneous generation of multiple proteins. An expressed protein is purified by affinity binding between the 6×His tag and the nickel-nitrilotriacetic acid (Ni^2+^-NTA) magnetic beads.

We demonstrated that CFPS could simultaneously synthesize multiple proteins from PCR-generated coding sequences within 6 h. CFPS bypassed some time-consuming procedures that require gene cloning and protein expression using a host cell, such as E. coli. One critical step of CFPS is to prepare a formatted target coding sequence, which involves two rounds of PCR amplification to add the adaptor sequences (containing the essential gene transcription and translation elements) (see Fig. S1 in the supplemental material). From 32 coding sequences randomly selected from 14,962 predicted ORFs of *Py. insidiosum* (8, 9), 24 target sequences (10 full-length and 14 partial genes with sizes ranging from 0.5 to 2.9 kb) were successfully prepared from gDNA to serve as the protein expression templates (Fig. S2; Table S1). Of these 24 templates, CFPS can synthesize 18 expected proteins (Fig. 3), which accounted for a 75% success rate. Western blot and LC-MS/MS analyses confirmed the identity and fidelity of each synthesized protein (Fig. S3). One round of CFPS synthesized up to 16 proteins with concentrations ranging from 92 to 387 μg/ml (Table S1). The obtained amount of protein was adequate for an initial biochemical and immunological characterization. A relatively small amount of several contaminated proteins appeared together with the synthesized protein, positive control (GFP), and even negative control (no coding sequence template). These contaminations could be histidine-rich proteins (presented in the E. coli extract used in CFPS) that can bind the affinity Ni^2+^-NTA magnetic beads during the purification step.

In search of a protein candidate (i.e., drug and vaccine target) for the development of an efficient diagnostic or therapeutic method for pythiosis, we initially screened all obtained synthesized proteins against a set of pythiosis (*n* = 5) and control (*n* = 3) sera. Dot blot analysis showed that most (67%) of the synthesized proteins did not react with any of the pythiosis sera tested (Fig. 3A). These proteins may not trigger host immunity during the *Py. insidiosum* infection, or their structures and biochemical properties were altered from their natural forms. Five synthesized proteins (IDs, 1, 4, 5, 11, and I25) reacted with some, but not all, pythiosis sera, indicating that they were either unequally recognized by host immunity or differently expressed among *Py. insidiosum* strains. Only the synthesized I06 protein was strongly recognized by all pythiosis sera, but not the control sera tested (Fig. 3A and B). The immunoreactivity of the I06 protein was confirmed by ELISA and an extended number of pythiosis (*n* = 21) and control (*n* = 25) sera from humans and various animals. The pythiosis sera showed significantly higher immunoreactivity (4.3-fold) against the I06 protein compared with the control sera (Fig. 3C). Taken together, I06 was a prominent immunoreactive protein of *Py. insidiosum* that might be a potential diagnostic and therapeutic target.

The established serodiagnostic tests for pythiosis rely on the use of crude protein extract (i.e., SABH and CFA) for the detection of anti-Py. insidiosum antibodies in patient sera (7, 38–43). Based on our experiences, the production of the crude protein extract exhibits a batch-to-batch biological variation (i.e., protein concentration and composition). Besides, it contains multiple protein species that could cross-react with the antibodies against proteins of other pathogens. For these reasons, the use of crude protein extract leads to concern on the limited reproducibility and detection specificity of such serodiagnostic tests. Since I06 is an oomycete-specific protein, it could prove to be a useful marker for the development of a more reliable and efficient test for the diagnosis of pythiosis. Regarding treatment, the current form of vaccine has been prepared from the crude protein extract of Py. insidiosum (4, 5, 7). Because of its limited immunotherapeutic efficacy (4, 5, 44), a novel protein candidate is urgently needed for the development of a better vaccine. As shown here, the I06 protein can strongly stimulate the host immune response, and its homolog is absent in humans, making it an appealing vaccine candidate. Future *in vivo* experiments in an animal model (45) could provide more information on the potential use of the I06 protein as a vaccine for the management of pythiosis.

We explored the function and cellular location of the I06 protein. Conserved domain analysis (26–29) identified a signal peptide and several domains, including the TOS1-like glycoside hydrolase domain, which contains an essential motif exhibiting laminarinase activity (Fig. 4A). The I06 protein architecture is compatible with the typical characteristic of the secretory elicitor protein, called OPEL (30). The OPEL-like I06 protein of *Py. insidiosum* can stimulate host antibody responses (Fig. 3A to C), similar to the OPEL protein of the plant-pathogenic oomycete *P. parasitica* that triggers plant defense mechanisms (30). Biochemical analysis of the synthesized I06 protein did not show any glycoside hydrolase activity against the substrates tested (i.e., cellulose and laminarin) (Fig. 3D). The absence of such a biochemical property of the I06 protein may be due to the lack of essential folding and posttranslational modifications (which are drawbacks of expressing a eukaryotic protein under the prokaryotic conditions) or the lack of exon 1 expression (which resulted in an incomplete protein; Fig. 4A). Mass spectrometric data generated from SABH (representing the cytosolic proteins) and especially CFA (representing the extracellular proteins) extensively mapped the I06 protein sequence (Fig. 2B to D), indicating that *Py. insidiosum* produced and secreted a significant amount of this protein.

Twenty-nine I06 homologs, found in 15 oomycetes (one to three homologs/species) (Fig. S4), had the typical structure that is compatible with the OPEL protein (30). The presence of the OPEL-like protein is unique to the oomycetes, as no homolog was identified in the other organisms (including humans). Regardless of the phylogenetic relationship among the oomycetes (Fig. 6), these oomycete-specific OPEL-like proteins can be divided into groups A (16 proteins) and B (13 proteins) (Fig. 5B). The OPEL-like proteins of nine oomycetes were in both groups, while the proteins of the other organisms were in either group A (four species) or B (two species) (Fig. 5B). The pairwise comparison supported the phylogenetic findings, as the protein sequence similarities within one group (up to 93%) were markedly higher than that between groups (up to 59%) (Fig. 5A). These results suggest that the presence and duplication of the OPEL-like protein-coding genes might occur in the common ancestor of the oomycetes before these genes underwent an independent evolution (i.e., gene gain, loss, and modification) during oomycete speciation. Some oomycetes possess two or three homologs of the I06-like protein. However, not all homologs could be transcribed or expressed by the organism. For example, two copies of the OPEL-like protein-coding genes (one each in groups A and B) were identified in *Py. insidiosum*. Only the group A homolog was expressed during hyphal growth. It is possible that the group B homolog of *Py. insidiosum* is nonfunctional or that its expression depends on a specific condition or developmental stage.

In conclusion, CFPS offers a fast *in vitro* multiprotein synthesis using the formatted DNA template (i.e., PCR product). It bypassed the gene cloning and expression steps required for the conventional host cell-dependent protein production. The E. coli lysate-based CFPS system used in this study came with a few limitations, such as: (i) restricted length of a target gene (less than 2 kb for PCR-based DNA template); (ii) lack of proper folding and modification; (iii) relatively small amount of obtained protein (based on our results: up to ∼100 μg/protein); and (iv) noticeable nonspecific protein contamination. Such technical limitations can be addressed as follows. (i) A plasmid DNA, instead of a PCR product, should be used to express a longer protein-coding sequence (longer than 2 kb). (ii) The posttranslational modification of a synthesized protein can be achieved with a eukaryotic cell extract-based CFPS system prepared from yeast, insect, or rabbit reticulocyte (18, 46–49). (iii) The baseline protein contamination from the cell extract-based CFPS can be minimized by using the PURE (protein synthesis using recombinant elements) CFPS system, which relies on purified recombinant transcription and translation components (14, 50, 51). As shown here, CFPS opens the door to an extensive functional study of an organism of interest in the postgenomic era. For example, CFPS successfully expressed 18 proteins of *Py. insidiosum*. One of these proteins, I06, was a secretory, OPEL-like, and prominent immunoreactive protein of *Py. insidiosum*. The I06 protein could be a suitable target for the development of a novel and efficient diagnostic test by replacing the use of crude protein extract (i.e., SABH and CFA) as the antigen source in most established serological assays for pythiosis (20, 39–43, 52, 53). The I06 protein is also a potential target for developing a therapeutic vaccine, as it showed strong immunoreactivity against pythiosis patients.

## MATERIALS AND METHODS

### Ethics.

This study was approved by the Committee for Research, Faculty of Medicine Ramathibodi Hospital, Mahidol University (approval numbers MURA2019/691 and MURA2020/122).

### Microorganism and crude protein extraction.

The Pythium insidiosum Pi-S strain was subcultured on Sabouraud dextrose (SD) agar for 1 week. Several small pieces of agar-attaching hyphae were transferred to a 250-ml flask containing 100 ml of SD broth for shaking incubation (∼100 rpm) at 37°C for 7 days. The hyphal material was removed by filtration through a 0.2-μm-pore-size membrane (Merck Millipore, USA). The harvested hyphae were extracted to obtain SABH, as previously described (24, 54). The cell-free culture broth was concentrated using an Amicon centrifugation tube (10,000 molecular weight cutoff [MWCO]; Merck Millipore) to obtain CFA, according to the reported protocol (24, 54). Both SABH and CFA were stored at –30°C until use.

### Gene selection and primer design.

A total of 32 candidate genes were randomly selected from the list of 14,962 predicted ORFs of *Py. insidiosum* (8, 9), without prior knowledge of gene length, number of exons or introns, functional annotation, codon usage, or any other predicted biochemical property. Twelve genes contain no intron, and their full-length ORFs were used for cell-free protein synthesis. In contrast, the other 20 genes comprise at least 1 intron, and the selected exons for protein expression were shown in Table S1 in the supplemental material. A pair of 18-base-long primers were designed to amplify each selected protein-coding sequence from gDNA. All gene-specific forward primers (assigned as 1F) were linked with a 21-base adaptor sequence containing a start codon (ATG) and a polyhistidine tag (6×His) (Fig. S1A). In contrast, all gene-specific reverse primers (assigned as 1R) were attached to a 21-base adaptor sequence containing a stop codon (TAG) (Fig. S1A). These customized primers were purchased from Bioneer (Daejeon, Republic of Korea).

### Amplification of target genes.

gDNA was extracted from *Py. insidiosum* strain Pi-S using an established protocol (55). The obtained gDNA served as a template in the first-step PCR amplification using the corresponding gene-specific primers (Table S1; Fig. S1A) and an ExiProgen ProXpress PCR template kit (Bioneer). Briefly, gDNA (10 ng), forward and reverse primers (10 pmol each), and nuclease-free water were mixed to the final volume of 20 μl in a lyophilized premix tube from the kit. PCR was performed in a Mastercycler Nexus gradient thermocycler (Eppendorf, Germany), using the following conditions: an initial denaturation at 94°C for 5 min, 30 cycles with 1 cycle consisting of denaturation at 94°C for 30 s, annealing at 58°C (or an optimal temperature) for 30 s (Table S1), and elongation at 72°C for 90 s, and a final elongation at 72°C for 5 min. A PCR product (5 μl) was mixed with the Fluorodye DNA fluorescent loading dye (1 μl) (SMOBIO, Taiwan) before separation by 1.5% agarose gel electrophoresis at 100 V for 30 min. The GeneRuler 100 bp Plus DNA Ladder (Thermo Scientific) served as molecular markers. The separated PCR product was visualized using a Gel Doc XR+ gel documentation system machine (Bio-Rad, CA, USA) and purified using an AccuPrep PCR/gel purification kit (Bioneer).

Each purified PCR product (10 ng) served as a template in the second-step PCR amplification (Fig. S1B). The ExiProgen ProXpress PCR template kit reagents, 5 ng each of the N-terminal upstream (harboring T7 promoter, ribosome binding site [RBS], and 6× histidine) and downstream (harboring T7 terminator) cassettes, 10 pmol each of the second-set forward (2F) and reverse (2R) primers (provided by the kit), and nuclease-free water were mixed in a 20-μl PCR mixture (Fig. S1B). PCR amplification was conducted in a Mastercycler Nexus gradient thermocycler (Eppendorf, Germany), using the following conditions: an initial denaturation at 94°C for 5 min, 30 cycles with 1 cycle consisting of denaturation at 94°C for 1 min, annealing at 48°C for 1 min, and elongation at 72°C for 90 s, and a final elongation at 72°C for 5 min. The obtained PCR product (Fig. S1C) was separated, visualized, and purified as described above. DNA concentration and purity were assessed using a NanoDrop 2000 spectrophotometer (Thermo Scientific).

### Automated cell-free protein synthesis.

Up to 16 proteins per run were simultaneously expressed and purified using an ExiProgen EC protein synthesis kit (Bioneer) and an ExiProgen automated cell-free protein synthesis machine (Bioneer). The manufacturer recommends adjusting the total amount of the second-step PCR product of each gene (that served as the protein synthesis template) according to its size, i.e., 0.5 μg for sizes less than 1 kb and 1 μg for sizes between 1 and 2 kb. As an exception, we increased the PCR product amount (up to 2 μg) of a few genes (i.e., IDs 5 and 10) to obtain a better protein yield. In short, 0.5 to 2 μg of each second-step PCR product was incubated with the E. coli extract and the kit master mix containing all components required for protein expression (i.e., NTPs, amino acids, and salts). The protein synthesis reaction was conducted at 30°C for 3 h. The resulting protein was subsequently purified using affinity interaction of 6×His and Ni-NTA magnetic nanoparticles at room temperature for 3 h. The protein concentration of each purified sample (in 250-μl volume) was measured using the Protein Assay reagent (Bio-Rad), following the manufacturer’s protocol.

### SDS-PAGE and Western blot analysis.

A purified protein sample (15 μl) was mixed with 5 μl of the protein loading dye (0.3 M Tris-HCl, 0.6 M dithiothreitol [DTT], 10% SDS, 0.06% bromophenol blue, and 30% glycerol) and boiled for 5 min. The protein-dye mixture (10 μl) was separated by using 12% SDS-PAGE (30% Bio-Rad acrylamide/bis-acrylamide 37.5:1) and a Bio-Rad Mini-Protean Tetra system (setting, 80 V for 90 min). The Precision Plus Protein Kaleidoscope Prestained Protein Standard (Bio-Rad) served as molecular weight markers. Separated proteins were blotted onto a 0.45-μm nitrocellulose membrane (Bio-Rad), using the Bio-Rad Mini-Protean Tetra system.

For immunodetection of a 6×His-tagged protein, the membrane was blocked with 5% skim milk in Tris-buffered saline with Tween 20 (TBS-T) (20 mM Tris, 150 mM NaCl and 0.1% Tween 20; pH 7.6) for 1 h and then incubated with the mouse anti-6×His monoclonal antibody (Abcam, Cambridge, UK; 1:5,000 in TBS-T with 1% skim milk) at 4°C overnight. After the membrane was washed three times with TBS-T, goat anti-mouse IgG (H+L) conjugated with horseradish peroxidase (HRP) (Bio-Rad; 1:5,000 in TBS-T with 1% skim milk) was added to the membrane, which was allowed to react at room temperature for 2 h. The membrane was washed three times before addition of the substrate solution (10 μl of 30% H_2_O_2_, 50 μl of 10% CoCl_2_, and 10 μl of 3,3′-diaminobenzidine tetrahydrochloride [DAB] in 10 ml phosphate-buffered saline [PBS]). The reaction was stopped by washing the membrane in distilled water.

For assessing protein immunoreactivity, the blotted membrane was blocked with 5% skim milk in TBS-T at room temperature for 1 h, incubated with each serum sample (1:2,000 in TBS-T with 1% skim milk) from pythiosis patients (*n* = 5) and healthy blood donors (*n* = 3) at room temperature for 3 h, and washed with TBS-T three times. The membrane was treated with the HRP-conjugated goat anti-human IgG antibody (Bio-Rad; 1:40,000 in TBS-T with 1% skim milk) for 1 h before proceeding to signal development using DAB, as described above.

### Mass spectrometry-based validation of the synthesized proteins.

Each synthesized protein was separated and excised from an SDS-PAGE gel stained with 0.1% brilliant blue R dye (Sigma, MO, USA). The isolated protein was digested with 0.1 mg/ml trypsin and then processed for LC-MS/MS analysis using an Ultimate 3000 nano-LC system (Dionex, Surrey, UK), following the reported protocol (9). The obtained mass spectrometric data (in the “.mgf” file format) were searched against the in-house Mascot library of 14,962 *Py. insidiosum*’s proteins, as described by Rujirawat et al. (9).

### Dot blot.

Each synthesized protein sample (1 μl) was spotted onto a nitrocellulose membrane and air dried overnight. The blotted membrane was blocked with 5% skim milk in TBS-T for 1 h, incubated with each serum sample (1:2,000 in TBS-T with 1% skim milk) from pythiosis patients (*n* = 5) and healthy blood donors (*n* = 3) at room temperature for 3 h, and washed three times with TBS-T. The membrane was then incubated with the HRP-conjugated goat anti-human IgG antibody (Bio-Rad; 1:40,000 in TBS-T with 1% skim milk) for 1 h. The membrane was washed three times before developing a signal using the DAB substrate as described above.

### ELISA.

Protein A/G-based ELISA was performed by adapting the protocol of Jaturapaktrarak et al. (20, 38). In brief, a 96-well polystyrene plate (Corning, New York, USA) was coated with the I06 protein (0.1 μg/well; diluted in 0.1 M carbonate buffer) and incubated at 4°C overnight. All unbound proteins were removed by washing with 100 μl/well of phosphate-buffered saline (PBS) (137 mM NaCl, 2.7 mM KCl, 10 mM Na_2_HPO_4_, 1.8 mM KH_2_PO_4_; pH 7.4) four times and then washing with 250 μl/well of blocking buffer (0.5% bovine serum albumin in PBS) four times. The plate was blocked with 250 μl/well of the blocking buffer at 37°C for 1 h before washing four times with 100 μl/well of PBS with 0.1% Tween 20 (PBS-T). A serum sample (1:1,600 in PBS; 100 μl) from human and animal patients with pythiosis (15 humans, 4 horses, and 2 dogs) and control individuals with no sign of pythiosis (17 humans, 4 horses, 2 dogs, and 1 cat, and 1 cow) was added to each well (in duplicate) and incubated at 37°C for 1 h. The plate was washed with PBS-T (as described above) and incubated with the HRP-conjugated recombinant protein A/G (Thermo Scientific, MA, USA; 1:100,000 in PBS) at 37°C for 1 h. After another washing step, an ELISA signal was developed in the dark, using a 3,3′,5,5′-tetramethylbenzidine (TMB) substrate kit (Thermo Scientific). The reaction was stopped by adding 100 μl of 0.5 N sulfuric acid to each well. An optical density (OD) was measured at 450 nm, using an Infinite200 Pro ELISA plate reader (Tecan, Austria). Differences in the ODs from the pythiosis (*n* = 21) and control (*n* = 25) sera were assessed using the independent *t* test with 95% confidence (the PASW statistics software version 18, Statistical Package of Social Sciences, Inc., IL, USA). Graphs were created using 2016 Microsoft Excel (Microsoft Corporation, WA, USA).

### Glycolytic activity assay.

The I06 protein was assessed for glycoside hydrolase activity by using an established agar plate method (23, 24) with some modifications. Briefly, 90 μl of either the I06 protein (450 μg/ml), *Trichoderma harzianum* lysing enzyme (Sigma; 450 μg/ml) (positive control), Trichoderma reesei cellulase enzyme (Sigma; 450 μg/ml) (positive control), or distilled water (negative control) were spotted onto three 6-mm-diameter antibiotic assay discs (Whatman, GE Healthcare Life Sciences, UK). The paper discs were then placed on a 1.5% agar plate containing 1.5% Avicel PH-101 (Fluka-Sigma) diluted in 0.1 M sodium acetate buffer (pH 4.8) or a 1.5% agar plate overlaid with 1% laminarin diluted in sterile H_2_O (Sigma). The plates were incubated at 37°C for 24 h, flooded with 5 ml of Gram’s iodine solution (2 g KI and 1 g iodine in 300 ml distilled water) for 5 min, and destained with distilled water to observe a clear zone.

### Bioinformatic analysis of the I06 protein homologs.

Genome and transcriptome data of Py. insidiosum (8, 21) and gene model prediction (9) defined the full-length coding sequence of I06 protein (NCBI accession number GAX94098.1). A search of the I06 protein homologs was performed using the BLAST program against two public databases: NCBI (https://blast.ncbi.nlm.nih.gov/) and FungiDB (https://fungidb.org/). The presence of the homologous sequences was also checked in the genomes of 20 oomycetes and 2 diatoms, using the Oomycete Gene Table (25). Conserved domains of the I06 protein homologs were identified using the NCBI Conserved Domain Search Tool (26–29). Protein properties (i.e., such as molecular mass, isoelectric point [pI], and instability index) were calculated by the ProtParam program (56, 57). Posttranslational modification and signal peptide were predicted using ScanProsite (58) and SignalP 5.0 (59), respectively. Schematic illustrations of gene and protein structures were generated by using the DOG/IBS software (60, 61).

### Phylogenetic analysis and all-against-all pairwise sequence comparisons.

A phylogenetic analysis was conducted to understand the relationship between I06 homologous proteins (*n* = 29). This process started with an alignment of I06 homolog sequences using ClustalW version 2.1 with default parameters (62). The resulting multiple-sequence alignment result was then subjected to gap removal and subsequently used to create a maximum likelihood tree with bootstrap analysis using FastTree version 2.1.9 with default parameters (Jones-Taylor-Thornton [JTT]+CAT model) (63). The gap-removed multiple-sequence alignment result of I06 homologs was also used for an all-against-all pairwise sequence comparison. All possible pairs of I06 homolog sequences were extracted from the multiple-sequence alignment and compared to determine their sequence identities. The result was then visualized in a tabular form where the order of I06 homologs in rows and columns is based on the phylogenetic analysis result.

A protein-based phylogenetic analysis was also conducted to summarize the evolutionary relationship between 20 oomycetes and 2 diatoms (served as outgroups). The Oomycete Gene Table (25) was used to identify 14 single-copy core proteins found across 20 oomycete and 2 diatom genomes. A multiple-sequence alignment was created for each core protein using ClustalW version 2.1 with default parameters (62). Gaps were removed from each alignment result, and all of the resulting alignments were joined to create a concatenated multiple-sequence alignment of core proteins (6,169 amino acids in length). FastTree version 2.1.9 was then employed to create a maximum likelihood tree with bootstrap analysis using default parameters (JTT+CAT model) (63). Finally, FigTree version 1.4.3 (http://tree.bio.ed.ac.uk/software/figtree/) was used to visualize all phylogenetic tree results.

### Data availability.

The NCBI accession numbers (https://www.ncbi.nlm.nih.gov) of all 32 candidate genes/proteins, used for cell-free protein synthesis, are consolidated in Table S1. The LC-MS/MS data, used for the validation of 18 synthesized proteins, are available in Data Set S1 in the supplemental material.

10.1128/mSystems.00196-20.6 DATA SET S1 LC-MS/MS data used for the validation of 18 successfully synthesized proteins. Download Data Set S1, XLS file, 0.6 MB. Copyright © 2020 Sae-Chew et al. 2020 Sae-Chew et al. This content is distributed under the terms of the Creative Commons Attribution 4.0 International license.
