# Supplementary material for: Automated Cell-Free Multiprotein Synthesis Facilitates the Identification of a Secretory, Oligopeptide Elicitor-Like, Immunoreactive Protein of the Oomycete Pythium insidiosum
Source: mSystems. 2020 May 12;5(3):e00196-20. doi: 10.1128/mSystems.00196-20 (PMC7219551; doi:10.1128/mSystems.00196-20)
Supplement: TABLE S1 [file mSystems.00196-20-st001.pdf]

ID	Functional annotation	Accession number	Protein validation by LC-MS/MS	Coding sequence length (bp)	Number of intron(s)	Expressed sequence	Gene-specific primer sequences		1st-step PCR annealing (°C)	Final amplicon		Synthetic proteins				
							Forward	Reverse		Size (bp)	Concentration (ng/µL)	Length (amino acids)	Size (kDa)	Concentration (µg/mL)	Yield (µg)	Mapped peptides
1	Hypothetical protein	GAY00614.1	Yes	525	-	Full length	5'-CTTACATTAGTTGTCCAC-3'	5'-GCGCACGAGGTACGCGCT-3'	58	761	72	174	20.29	145.7	36.43	12
2	Protease inhibitor Epi11	GAX97702.1	Yes	642	-	Full length	5'-TGCACACAAGACTACACG-3'	5'-GTGACGTAGGCGCTTGCA-3'	60	-	-	-	-	-	-	-
3	Elongation factor-like, partial	GAX97496.1	Yes	882	-	Full length	5'-GACTCGTGCGACTGGTCG-3'	5'-AGCCTTGTACTCGACGGC-3'	58	1,118	84.4	293	33.78	223.7	55.93	23
4	Hypothetical protein	GAX94187.1	Yes	1,254	-	Full length	5'-ATGTGCCACAAGTACATG-3'	5'-AACGCAGTGCTTGCCAGG-3'	56	1,490	95.1	417	45.66	218.9	54.73	10
5	Translation elongation factor alpha	GAY03155.1	Yes	1,368	-	Full length	5'-GCGGGTGAAAAGCAGCAT-3'	5'-AGCCTTGTACTCGACGGC-3'	58	1,604	107.3	455	51.25	193.5	48.38	8
6	Eukaryotic translation initiation factor 3	GAY02261.1	Yes	2,088	-	Full length	5'-GCGCCACCGTCGTTCGAG-3'	5'-CTTGCGCACGACCTCCTC-3'	58	2,324	103.1	695	81.09	167.5	41.88	9
7	Transketolase	GAY02637.1	No	2,097	-	Full length	5'-GCTGAGCACGTCGACAAG-3'	5'-GTGGCCGTGCGCCGACGC-3'	62	-	-	-	-	-	-	-
8	Exo-1,3-beta-glucanase	GAX98002.1	No	2,211	-	Full length	5'-CTGCGCTCTCGGACATGG-3'	5'-TGGGCGGCCGATGGGGTT-3'	58	2,447	90.7	736	82.66	92.3	23.08	5
9	Methyltransferase	GAX96865.1	Yes	2,346	-	Full length	5'-ACCGTCGTCGCCTCGACC-3'	5'-CTGCTGCTGCAGCTTGGC-3'	58	2,582	91.1	781	87.94	156.4	39.10	21
10	Aconitate hydratase, mitochondrial	GAX99121.1	Yes	2,346	-	Full length	5'-ATCGCCCTCTCCGCTGCG-3'	5'-AGCCGAGTTCTTCATCTT-3'	58	2,582	116.3	781	85.28	203.3	50.83	37
11	Chaperone DnaK	GAX92393.1	Yes	2,364	-	Full length	5'-GCCCCGAAGCGTCGCAGC-3'	5'-GAAGCGGATCATGCGCTC-3'	58	2,600	95.5	787	87.72	248	62.00	30
13	Glycine dehydrogenase	GAX97563.1	Yes	2,856	-	Full length	5'-CTCGCCGCCGTCGGGTTC-3'	5'-CTCGTAGTCCGTGAGTGG-3'	58	3,092	77.4	-	-	-	-	-
I01	Hypothetical protein	GAX96072.1	Yes	969	1	Exon1	5'-AAGTTCCTCGCCCTCGCC-3'	5'-AGAACAAGGGCAGCGACC-3'	-	-	-	-	-	-	-	-
I02	Hypothetical protein	GAX93918.1	Yes	975	1	Exon2	5'-GAGGTCGACGTGGTTCTC-3'	5'-GTCGCTCTTCGTCTCGCA-3'	58	731	84.9	164	18.59	230.5	57.63	4
I03	Coatomer subunit beta'-2-like protein	GAY03339.1	No	1,017	1	Exon2	5'-ACGTCCACCGTCGCTACG-3'	5'-GAGTCGCGCGATCTTGTC-3'	-	-	-	-	-	-	-	-
I04	Glycoprotein elicitor precursor	GAX93925.1	Yes	1,044	1	Exon1	5'-TCCCCCACGACGTTCGCC-3'	5'-GTAGAGCAGGCTCTTGGC-3'	-	-	-	-	-	-	-	-
I05	ATP synthase subunit beta, mitochondrial	GAX95749.1	Yes	1,509	1	Exon2	5'-GTCCCTGTCGGTCCGGAG-3'	5'-CTCGTCTAATTCGGCCGC-3'	58	1,442	76.9	401	43.69	189.7	47.43	5
I06	Putative GPI anchor protein	GAX94098.1	Yes	1,719	1	Exon2	5'-GTCGAGTTCTCTATCACG-3'	5'-CAGCGACTTCTTGTCAGA-3'	58	1,730	105.6	497	54.68	152	38.00	12
I07	Hypothetical protein	GAX99152.1	Yes	2,203	1	Exon1	5'-ACGCTTCAGTATCCCTCC-3'	5'-CTTGGTCTCGCGGAAAAG-3'	58	1,568	103.2	443	50.7	387.1	96.78	28
I08	Hypothetical protein	GAX93797.1	Yes	2,457	1	Exon2	5'-CGCGTTTCTCGATGGCGT-3'	5'-GACGACGGGGGCGATGAG-3'	58	2,288	101.6	-	-	-	-	-
I09	Putative alpha amylase	GAX96590.1	Yes	2,619	1	Exon2	5'-CTCCTTGAACTCGTACAC-3'	5'-GCAGGTATTCGGCAGTCC-3'	58	1,142	96.4	-	-	-	-	-
I11	Hypothetical protein	GAX97202.1	Yes	2,913	2	Exon1	5'-CTTCCCAGCCTCGGCACC-3'	5'-ATCGCCATCCGACTTGCG-3'	58	1,028	96.7	263	30.23	380.4	95.10	5
I13	Serine protease family S33	GAX92657.1	Yes	5,808	3	Exon4	5'-ATGCTCCTCCCGCGCCGC-3'	5'-AACCTCGCCGTCAGCCGA-3'	62	-	-	-	-	-	-	-
I14	Ornithine aminotransferase, mitochondrial	GAX92425.1	Yes	1,329	4	Exon4	5'-CTCCCGATGAACACGGGT-3'	5'-GCGTTCATCTCCGCGCGG-3'	56	925	72.7	228	25.71	126.4	31.60	3
I16	Hypothetical protein	GAY01417.1	Yes	2,160	5	Exon4	5'-CGGATCCCTCCTCCTCCT-3'	5'-CTGGAGGGCAGCCATGAT-3'	58	860	95.4	207	23.7	197.6	49.40	6
I18	Hypothetical protein	GAX96238.1	Yes	5,556	5	Exon6	5'-AACAAGGACTTT GTGCTC-3'	5'-CTCGACGCTGCCAAACAC-3'	56	1,733	111.9	-	-	-	-	-
I19	Hypothetical protein	GAX96174.1	Yes	2,787	6	Exon6	5'-ATGGATGCCCTGCAGGCA-3'	5'-CTTGAGCAGCAGCTCCGC-3'	58	1,445	112.5	402	45.61	173.7	43.43	3
I20	ABC transporter E family member 2	GAY01773.1	Yes	3,504	6	Exon7	5'-GTCCGTGAAGGCATCAAC-3'	5'-ATCGTCCATGAAAAAGTA-3'	58	1,202	119	-	-	-	-	-
I21	Trans-acting enoyl reductase, putative	GAX92617.1	Yes	3,534	6	Exon6	5'-GACGGCATGGACGCGACG-3'	5'-CTCCGACGATCATCTTGC-3'	64	-	-	-	-	-	-	-
I22	Glucoamylase GlaM	GAX94165.1	Yes	3,783	7	Exon1	5'-GTGTTCCTCTCCGTCCAC-3'	5'-GCCGACAGCGACTTCAGC-3'	58	2,044	84.1	-	-	-	-	-
I24	Elongation factor 2	GAX93982.1	Yes	10,392	16	Exon3	5'-GTGAACTTCACGGTCGAT-3'	5'-AACGCTGAGAAGTGTGCC-3'	-	-	-	-	-	-	-	-
I25	Heat shock 70 kDa protein	GAX96752.1	Yes	6,474	20	Exon9	5'-CGCAAGCACCGCAAGGAC-3'	5'-CTCCTCGATCTTCGGGCC-3'	58	1,442	119.1	401	45.12	290.2	72.55	25
