# Supplementary figures and images for: Automated Cell-Free Multiprotein Synthesis Facilitates the Identification of a Secretory, Oligopeptide Elicitor-Like, Immunoreactive Protein of the Oomycete Pythium insidiosum
Source: mSystems. 2020 May 12;5(3):e00196-20. doi: 10.1128/mSystems.00196-20 (PMC7219551; doi:10.1128/mSystems.00196-20)

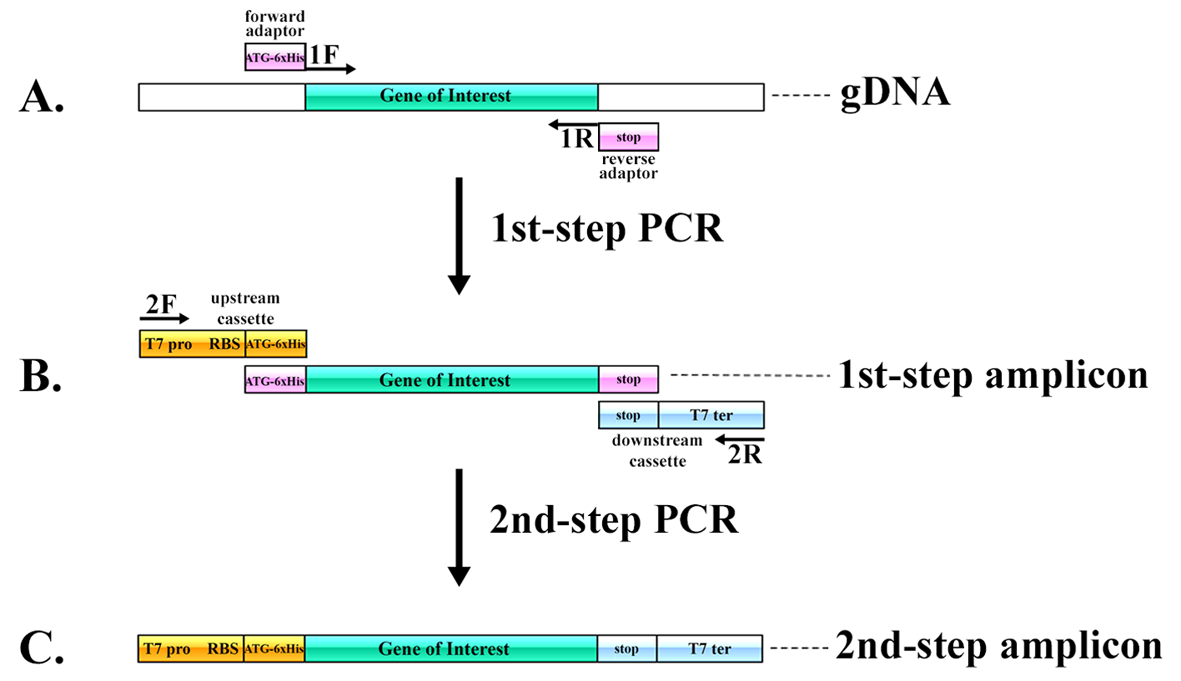

Supplement: FIG S1 [file mSystems.00196-20-sf001.tif]

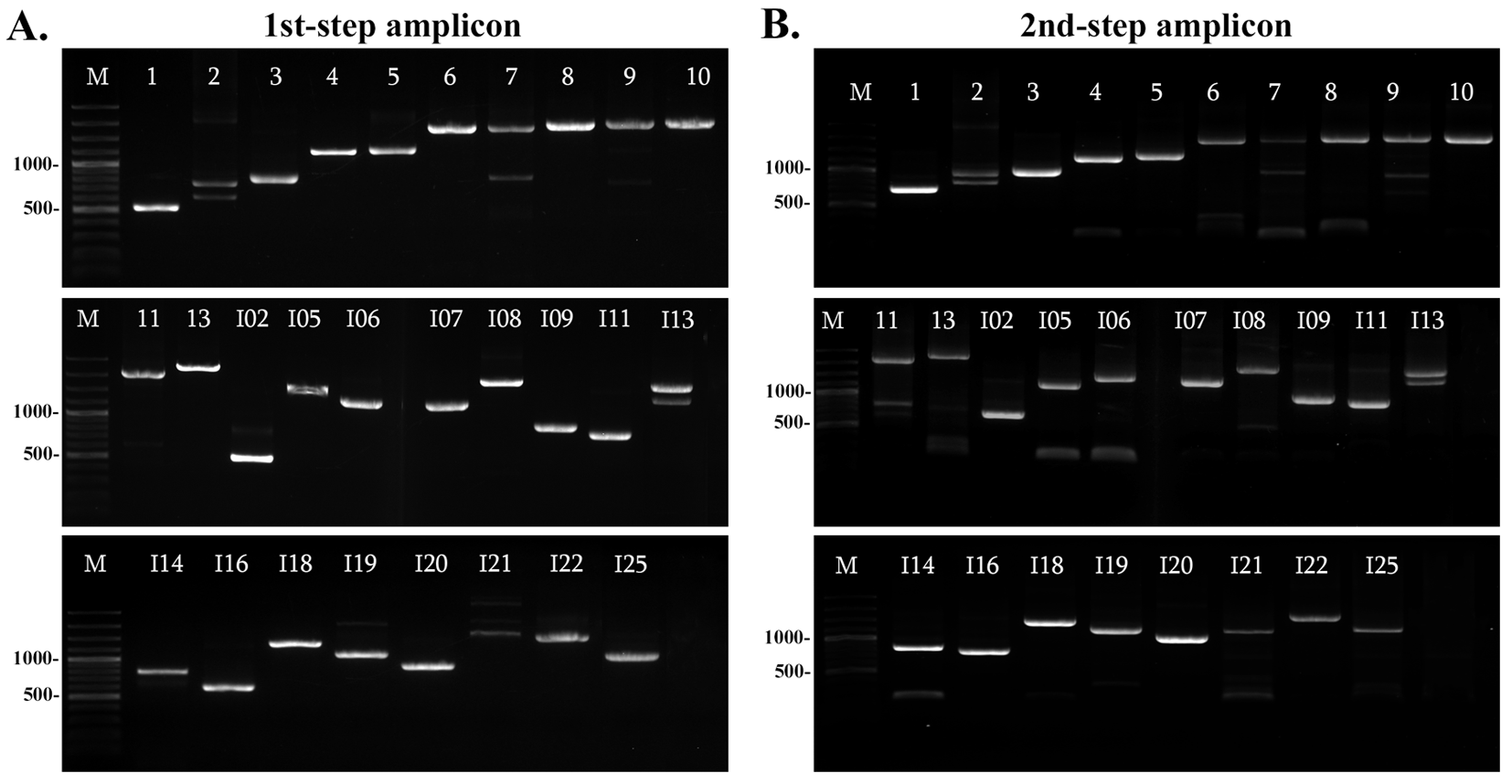

Supplement: FIG S2 [file mSystems.00196-20-sf002.tif]

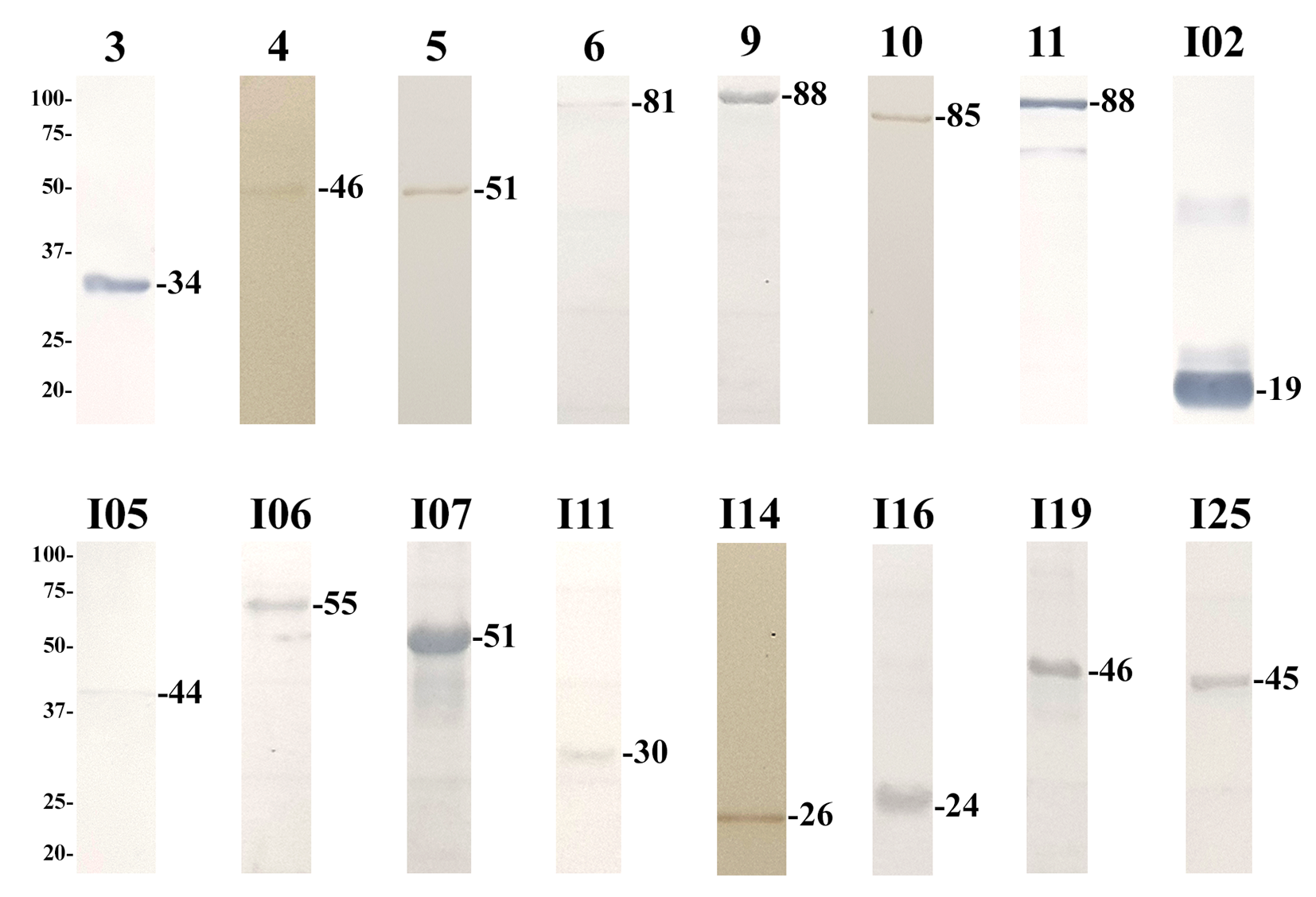

Supplement: FIG S3 [file mSystems.00196-20-sf003.tif]

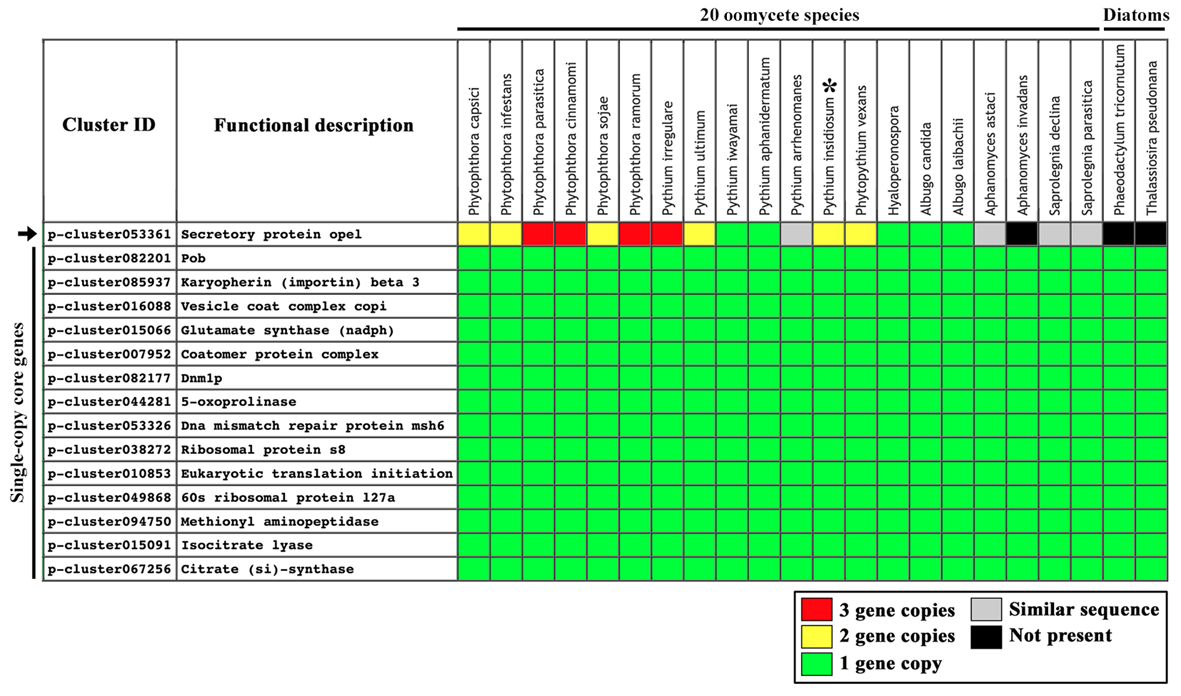

Supplement: FIG S4 [file mSystems.00196-20-sf004.tif]
